# Supplementary material for: Intercalation-type catalyst for non-aqueous room temperature sodium-sulfur batteries
Source: Nat Commun. 2023 Oct 17;14:6568. doi: 10.1038/s41467-023-42383-3 (PMC10582099; doi:10.1038/s41467-023-42383-3)
Supplement: Supplementary file 1 — Supplementary Information [file 41467_2023_42383_MOESM1_ESM.pdf]

# Intercalation-type catalyst for non-aqueous room temperature sodium-sulfur batteries

Jiarui He[1], Amruth Bhargav[1], Laisuo Su[1], Harry Charalambous[2], and Arumugam Manthiram[1,*]

[1]Materials Science and Engineering Program & Texas Materials Institute, The University of Texas at Austin, Austin, TX 78712, USA.

[2]X-ray Science DivisionAdvanced Photon Source, Argonne National Laboratory, 9700 S. Cass Avenue, ArgonneLemont, IL 60439, United States

*e-mail: manth@austin.utexas.edu.

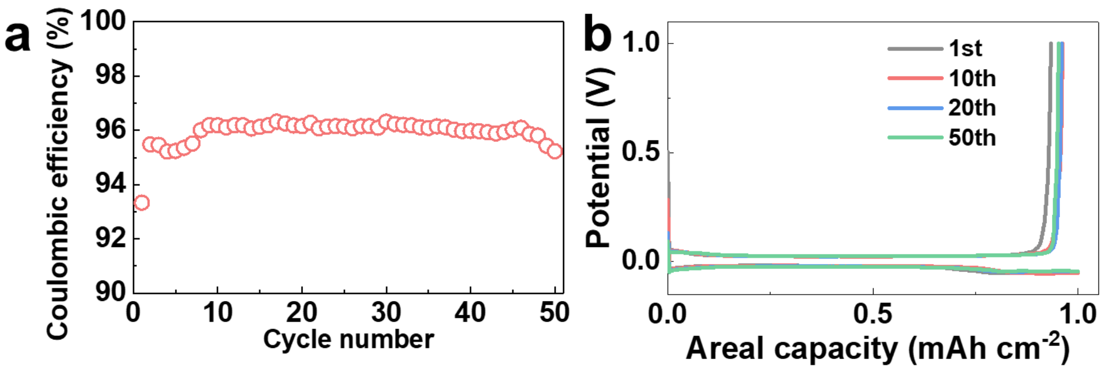


**Figure S1 |** (a) Coulombic efficiency of Na plating-stripping with Cu electrodes in LHCE at 1 mA $cm^{-2}$ with an areal capacity of 1 mAh $cm^{-2}$ and (b) the corresponding Na plating/stripping profile.

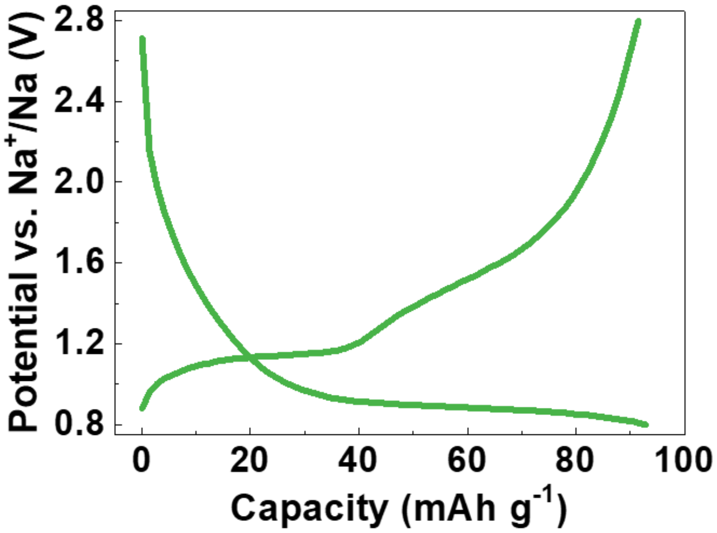


**Figure S2 |** Voltage profile of MTG||Na at 0.1C rate in the voltage range between 0.8 and 2.8 V, which can clearly show the intercalation in MTG.

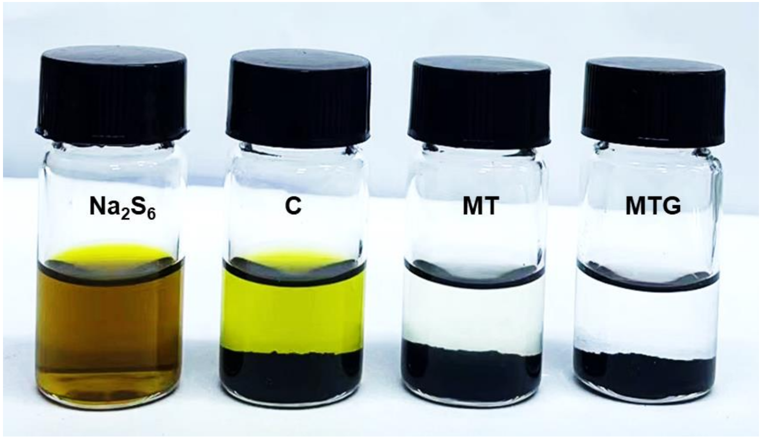


**Figure S3 |** Photograph of the sealed vials of a $Na_2S_6$/DME solution after contacting with C, pure $MoTe_2$ (MT), and MTG.

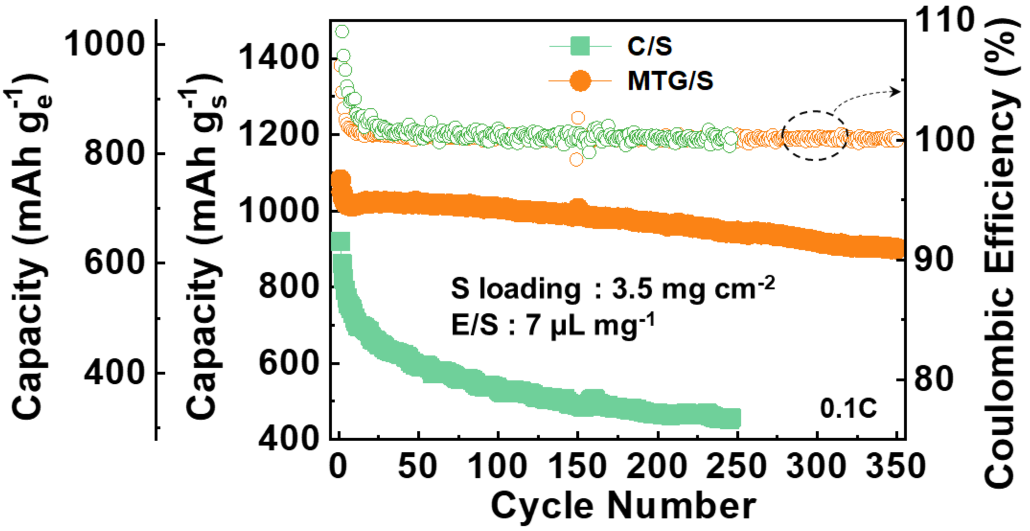


**Figure S4 |** Cycling performances of MTG/S cathode and C/S cathode at 0.1C rate. Here, as the sulfur content in each cathode is 70%, the capacity based on the total mass of electrode materials equals 70% of the capacity based on sulfur mass. The subscript e in the unit of mAh $g^{-1}_{e}$ means electrode.

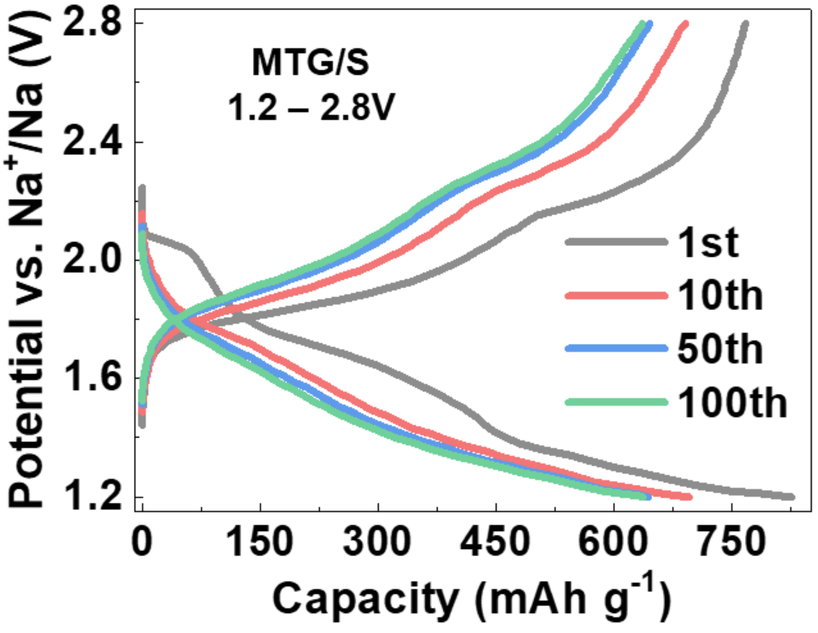


**Figure S5 |** Voltage profile of MTG/S in the voltage range between 1.2 and 2.8 V.

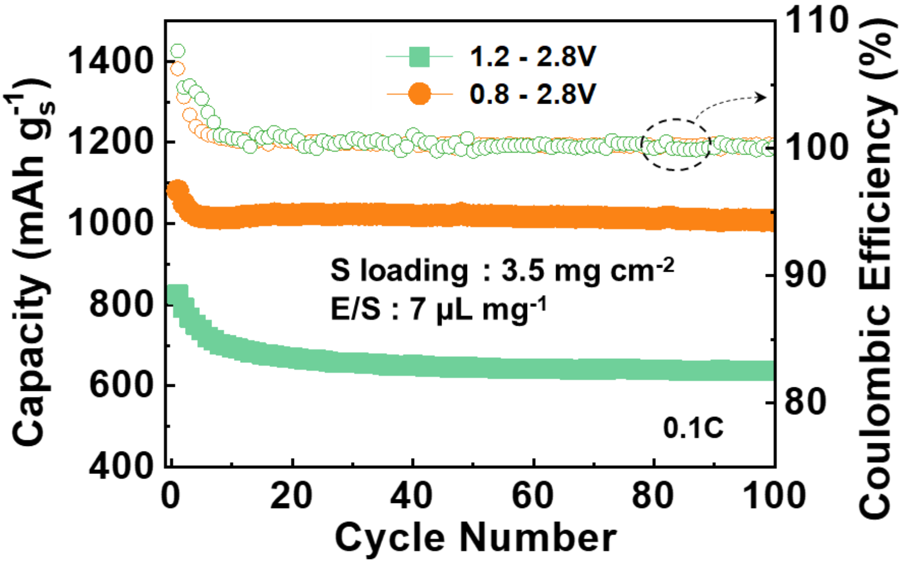


**Figure S6 |** Cycling performances of MTG/S cathode at 0.1C rate in different voltage ranges.

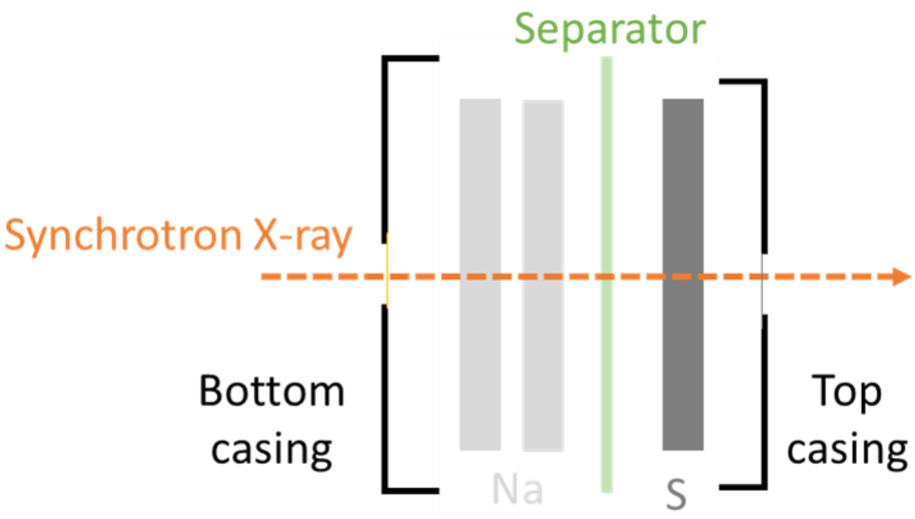


**Figure S7 |** Schematic of house-built CR2016-type window coin cells.

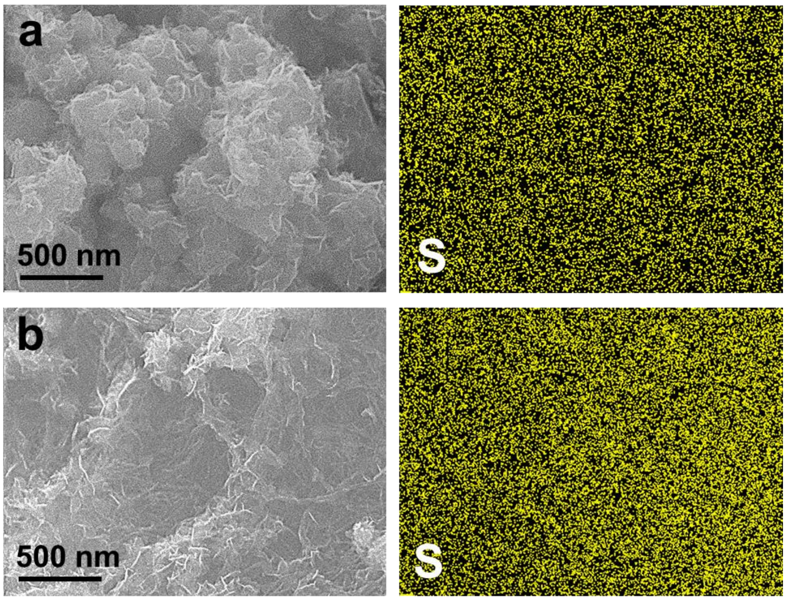


**Figure S8 |** SEM image of (a) MT/S and (b) MTG/S and their corresponding elemental mapping images of sulfur after 100 cycles.

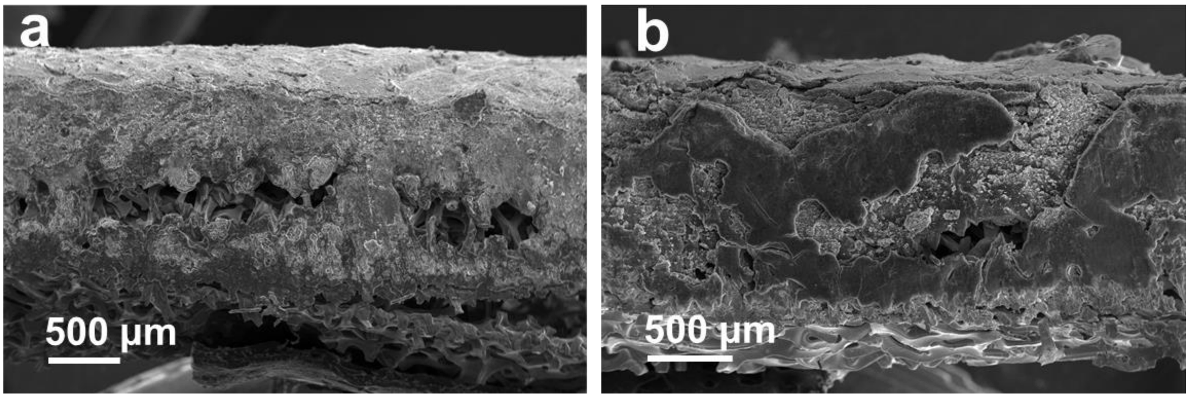


**Figure S9 |** Cross-sectional morphologies of Na in Na−S cells with (a) MTG and (b) C/S cathodes after 100 cycles at the 0.1C in the voltage window between 0.8V and 2.8V.

**Supplementary Table 1.** A comparative analysis of the Na-C cells with MTG/S and the state-of-the-art Na-S cells reported in the literature. (Note: C-rate is the charge/discharge current density, 1C = 1675 mA $g_s^{-1}$).

| Cathode | Electrolyte | S content/ loading in Electrode (wt.% / mg $cm^{-2}$) | E/S ratio (μL $cm^{-2}$) | Current density (C-rate) | Reversible capacity (mA h $g^{-1}$) | Cycle life | Capacity fading rate (%) | Reference |
|---|---|---|---|---|---|---|---|---|
| **MTG/S** | **LHCE** (DME : NaFSI : TTE = 1 : 1.2 : 1) | **69.6 / 3.5** | **7** | **0.1C** | **1081** | **350** | **0.05** | **This work** |
| | | **69.6 / 3.5** | **7** | **1C** | **799** | **900** | **0.02** | |
| | | **69.6 / 5.8** | **7** | **0.1C** | **887** | **100** | **0.26** | |
| C/S (Ketjen Black carbon/Sulfur) | LHCE | 69.6 / 2 | 15 | 0.1C | 922 | 300 | 0.09 | R1 |
| covalent-SC | $NaClO_4$ in EC/DEC/FEC | 26.6 / 1.4 | -- | 0.8C | 1,070 | 600 | 0.028 | R2 |
| S/C (sodiated hard carbon anode) | $NaClO_4$ +$Na_2S/P_2S_5$ TEGDME | 40.5 / 2.4 | 25 | 0.05C | 920 | 1000 | 0.06 | R3 |
| ACC-40S | $NaClO_4$ in EC/PC/FEC | 32.0 / 1 | -- | 0.1C | 1492 | 400 | 0.06 | R4 |
| CN/Au/S | $NaClO_4$ in PC/FEC | 39.6 / -- | -- | 0.06C | 701 | 110 | 0.58 | R5 |
| S/C | $NaClO_4$ +$Na_2S/P_2S_5$ TEGDME | 47.0 / 2 | -- | 0.05C | 780 | 70 | 0.88 | R6 |
| S@MPCF | NaTFSI in PC/FEC/$InI_3$ | 48.8 / 0.36 | 50 | 0.1C | 1170 | 500 | 0.011 | R7 |
| S/C | $NaClO_4$ + $NaNO_3$ in TEGDME | 38.4 / 2.5 | 7.5 | -- | 1000 | 20 | 3 | R8 |
| $Na_2S_6$–CC@$MnO_2$ | $NaClO_4$ + $NaNO_3$ in TEGDME | -- / 1.7 | -- | 0.12C | 938 | 150 | 0.16 | R9 |
| C/S/BTO | $NaClO_4$ in EC/DEC | 62 / 1.2 | -- | 0.3C | 1101 | 400 | 0.11 | R10 |

| | | | | | | | | |
|---|---|---|---|---|---|---|---|---|
| S/C | $NaPF_6$ in TEGDME | 40.0 / -- | -- | 0.1C | 776 | 20 | 1.7 | R11 |
| S@Ni-NCFs | MCS-Li | 36 / 0.7 | 128 | 1C | 431 | 270 | 0.17 | R12 |
| core−shell ZCS@S | $NaClO_4$ in EC/DEC/FEC | 45.6/2 | -- | 0.6C | 572 | -- | -- | R13 |
| $FeS_2$@NCMS/S | $NaClO_4$ in EC/PC/FEC | 45.9/-- | -- | 0.06C | 1471 | 300 | 0.2 | R14 |
| S/$MoS_2$/NCS | -- | 35/-- | -- | 0.6C | 427 | 2800 | 0.0055 | R15 |
| $Co_1$-ZnS/C@S | $NaClO_4$ in EC/DEC/FEC | 52/-- | -- | 0.6C | 620 | -- | -- | R16 |

## Supplementary References


1. He, J., Bhargav, A., Shin, W. & Manthiram, A. Stable Dendrite-Free Sodium − Sulfur Batteries Enabled by a Localized High-Concentration Electrolyte. J. Am. Chem. Soc. **143**: 20241-20248 (2021).
2. Yan, J. et al. An *in Situ* Prepared Covalent Sulfur − Carbon Composite Electrode for High-Performance Room-Temperature Sodium − Sulfur Batteries. *ACS Energy Letters*. **5**: 1307-1315 (2020).
3. Pampel, J., Dörfler, S., Althues, H. & Kaskel, S. Designing room temperature sodium sulfur batteries with long cycle-life at pouch cell level. *Energy Storage Materials*. **21**: 41-49 (2019).
4. Guo, Q. et al. Ultrastable Sodium − Sulfur Batteries without Polysulfides Formation Using Slit Ultramicropore Carbon Carrier. *Advanced Science*: 1903246 (2020).
5. Wang, N. et al. High-performance room-temperature sodium − sulfur battery enabled by electrocatalytic sodium polysulfides full conversion. *Energ. Environ. Sci.* **13**: 562-570 (2020).
6. Kohl, M., Borrmann, F., Althues, H. & Kaskel, S. Hard Carbon Anodes and Novel Electrolytes for Long-Cycle-Life Room Temperature Sodium-Sulfur Full Cell Batteries. Adv. Energy Mater. **6**: 1502185 (2016).
7. Xu, X. et al. A room-temperature sodium − sulfur battery with high capacity and stable cycling performance. Nat. Commun. **9** (2018).
8. Yu, X. & Manthiram, A. Capacity Enhancement and Discharge Mechanisms of Room-Temperature Sodium-Sulfur Batteries. *ChemElectroChem*. **1**: 1275-1280 (2014).
9. Kumar, A. et al. High-energy density room temperature sodium-sulfur battery enabled by sodium polysulfide catholyte and carbon cloth current collector decorated with $MnO_2$ nanoarrays. *Energy Storage Materials*. **20**: 196-202 (2019).
10. Ma, D. et al. New Strategy for Polysulfide Protection Based on Atomic Layer Deposition of $TiO_2$ onto Ferroelectric - Encapsulated Cathode: Toward Ultrastable Free - Standing Room Temperature Sodium − Sulfur Batteries. *Adv. Funct. Mater.* **28**: 1705537 (2018).
11. Seh, Z. W., Sun, J., Sun, Y. & Cui, Y. A Highly Reversible Room-Temperature Sodium Metal Anode. *ACS Central Science*. **1**: 449-455 (2015).

12. Guo, B. et al. Nickel Hollow Spheres Concatenated by Nitrogen‐Doped Carbon Fibers for Enhancing Electrochemical Kinetics of Sodium–Sulfur Batteries. *Advanced Science*. **7**: 1902617 (2020).
13. Liu, H. et al. Electrocatalyzing S Cathodes via Multisulfiphilic Sites for Superior Room-Temperature Sodium–Sulfur Batteries. *ACS nano*. 14: 7259-7268 (2020).
14. Yan, Z. et al. A high‐kinetics sulfur cathode with a highly efficient mechanism for superior room‐temperature Na–S batteries. *Adv. Mater.* 32: 1906700 (2020).
15. Wang, Y. et al. Tunable Electrocatalytic Behavior of Sodiated $MoS_2$ Active Sites toward Efficient Sulfur Redox Reactions in Room‐Temperature Na–S Batteries. *Adv. Mater*. 33: 2100229 (2021).
16. Liu, H. et al. Sustainable S cathodes with synergic electrocatalysis for room-temperature Na–S batteries. *J. Mater. Chem. A.* 9: 566-574 (2021).
